# Co-occurrence of pathogenic *CYP24A1* gene variants and primary hyperparathyroidism: a diagnostic challenge

**DOI:** 10.1093/jbmrpl/ziag164

**Published:** 2026-10-09

**Authors:** Simone Della Valentina, Laura Pierotti, Chiara Sardella, Anna Dal Lago, Maria Adelaide Caligo, Agnese Giamborino, Filomena Cetani

**Affiliations:** Department of Clinical and Experimental Medicine, University of Pisa, Pisa, 56126, Italy; Department of Clinical and Experimental Medicine, University of Pisa, Pisa, 56126, Italy; Endocrine Unit 2, University Hospital of Pisa, Pisa, 56124, Italy; Department of Clinical and Experimental Medicine, University of Pisa, Pisa, 56126, Italy; Laboratory of Molecular Genetics, University Hospital of Pisa, Pisa, 56126, Italy; Department of Surgical, Medical and Molecular Pathology and Critical Care Area, University of Pisa, Pisa, 56126, Italy; Department of Clinical and Experimental Medicine, University of Pisa, Pisa, 56126, Italy; Endocrine Unit 2, University Hospital of Pisa, Pisa, 56124, Italy

**Keywords:** primary hyperparathyroidism, hypercalcemia, CYP24A1, vitamin D metabolism, hypercalciuria, CKD

## Abstract

Hypercalcemia is mostly caused by primary hyperparathyroidism (PHPT) or malignancy. Less commonly, hypercalcemia results from vitamin D-dependent mechanisms. Pathogenic variants in *CYP24A1*, which encodes the 24-hydroxylase responsible for inactivating 1,25(OH)_2_D_3_, can lead to hypercalciuria, nephrolithiasis/nephrocalcinosis, and hypercalcemia, typically with suppressed PTH. When CYP24A1-related disease coexists with PHPT, their biochemical features may overlap, potentially masking a vitamin D catabolic defect and complicating postoperative interpretation of hypercalcemia. We report 2 unrelated patients with moderate-to-severe hypercalcemia in whom PHPT and *CYP24A1* variants coexisted. The first patient was a 26-yr-old man with longstanding nephrolithiasis who initially had hypercalcemia with an inappropriately normal PTH level and imaging consistent with a hyperfunctioning parathyroid lesion, treated with parathyroidectomy (PTx). Several years later, PTH-suppressed hypercalcemia and elevated 25(OH)D_3_ and 1,25(OH)_2_D_3_ concentrations was detected despite the absence of vitamin D supplementation. Genetic testing revealed a heterozygous pathogenic *CYP24A1* variant (p.Leu409Ser), consistent with partial 24-hydroxylase deficiency. The second patient was a 59-yr-old woman with severe PHPT requiring surgery and later experienced life-threatening hypercalcemia (19.8 mg/dL) requiring hemodialysis, marked calcium fluctuations, particularly after calcium and calcitriol exposure following a second surgery, nephrocalcinosis, and progressive renal impairment. Genetic testing identified compound heterozygous *CYP24A1* variants (p.Glu143del and p.Gln82Arg). These cases broaden the clinical spectrum of combined PHPT and CYP24A1-related disease and highlight that persistent or recurrent hypercalcemia after PTx, particularly when PTH is suppressed, may prompt evaluation for *CYP24A1* defects, with potential implications for vitamin D avoidance and long-term renal surveillance.

## Graphical abstract

**Graphical abstract:**
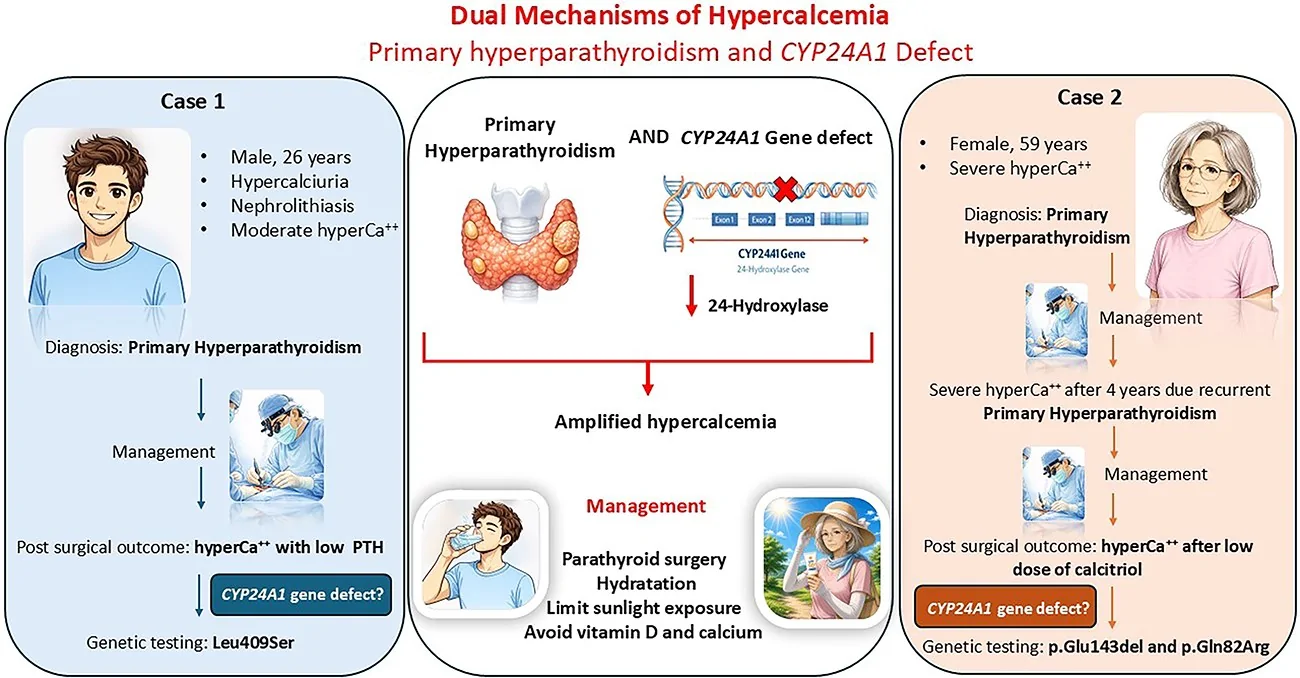

## Introduction

Hypercalcemia affects approximately 1% of the general population and is most commonly caused by primary hyperparathyroidism (PHPT) or malignancy, which together account for the majority of cases.^1^ Primary hyperparathyroidism is diagnosed when serum calcium is elevated on at least 2 occasions in the presence of elevated or inappropriately normal PTH levels. Management is guided by biochemical severity and end-organ involvement. Treatment options include parathyroidectomy (PTx), the only definitive therapy, or clinical and biochemical surveillance, according to the latest guidelines.^2^ In surgical candidates, preoperative localization studies are routinely performed to guide the operative approach, and post-operative follow-up is recommended to detect persistent or recurrent disease.^2^

A smaller proportion of hypercalcemia cases is attributable to vitamin D-dependent mechanisms, in which excess active vitamin D metabolites increase intestinal calcium absorption. These forms are usually acquired (eg, vitamin D intoxication, granulomatous disease, and selected malignancies) but can also reflect impaired vitamin D catabolism due to genetic defects.^3^ Pathogenic variants in the *CYP24A1* gene (cytochrome P450 family 24 subfamily A member 1), encoding the 24-hydroxylase responsible for vitamin D degradation, are a key cause. Biallelic loss-of-function *CYP24A1* variants were originally described as a cause of idiopathic infantile hypercalcemia-1 (OMIM 143880), but adult presentations, including in biallelic carriers, are increasingly recognized and may show substantial phenotypic variability.^4^ Monoallelic variants may present later in life with milder biochemical abnormalities, including hypercalciuria and high-normal calcium levels.^5^^,^^6^

Heterozygous carriers may display an intermediate biochemical phenotype and become clinically relevant when additional drivers of hypercalcemia are present.^5^^,^^7^ Coexistence with PHPT may amplify calcium and renal manifestations.^4^

Here, we report 2 unrelated patients harboring pathogenic *CYP24A1* variants and concomitant PHPT: one carrying a heterozygous variant and the other compound heterozygous for 2 pathogenic variants. We also review previously published reports of PHPT occurring in the setting of *CYP24A1*-related hypercalcemia.

## Materials and methods

### Assays

Venous blood samples were collected between 8:00 and 9:00 am following an overnight fast. Serum calcium, magnesium, phosphate, and creatinine were measured using standardized biochemical methods. Ionized calcium was assessed via ion-selective electrode methodology (Nova 8 calcium analyzer, Nova Biomedical). Plasma PTH was measured using a third-generation chemiluminescent immunoassay (DiaSorin LIAISON 1-84 PTH). Serum 25-hydroxyvitamin D [25(OH)D_3_] and 1,25(OH)_2_D_3_ were assessed by chemiluminescent immunoassay (IDS-iSYS, IDS plc; Liaison XL platform, DiaSorin). Bone turnover markers were measured using standard immunoassays. Reference ranges for biochemical parameters are provided in Table 1. Plasma intact FGF23 was assessed by immunochemiluminescent assay (Liaison XL platform, DiaSorin). Estimated glomerular filtration rate (eGFR) was calculated using the CKD-EPI (Chronic Kidney Disease Epidemiology Collaboration) formula.

**Table 1:** Biochemical parameters at baseline and after surgery of case 1.

Parameter	Pre surgery (July 2018)	50 mo after surgery (September 2022)	58 mo after surgery (May 2023)	Reference range
**Serum calcium (mg/dL)**	12.5	11.3	10.1	8.6-10.2
**Ionized calcium (mmol/L)**	1.76	1.41	1.32	1.13-1.32
**PTH (pg/mL)**	24	5.0	9.0	8-40
**Phosphate (mg/dL)**	2.1	3.0	2.9	2.5-4.5
**Magnesium (mg/dL)**	2.0	2.0	2.0	1.7-2.5
**eGFR (mL/min/1.73 m** ^**2**^ **)**	63	54.9	65	
**Creatinine (mg/dL)**	1.45	1.65	1.47	0.7-1.2
**BSAP (mcg/L)**	23	16	16	4.7-27.1
**Osteocalcin (mcg/L)**	46.6	16.4	16.4	6.8-34
**S-CTX (mcg/L)**	–	0.264	0.331	0.038-0.724
**FGF23 (ng/L)**	–	–	272	23.2-95.4
**24 h urinary calcium (mg/24 h)**	558	574	330	100-300
**25(OH)D_3_ (ng/mL)** ^**a**^	69.1	72.8	47.9	20-100
**1,25(OH)_2_D_3_ (pg/mL)** ^**a**^	–	64.9	45.6	20-79
**Vitamin D_3_ (ng/mL)** ^**b**^	–	4.35	1.34	
**25(OH)D_3_ (ng/mL)** ^**b**^	–	61.6	36.6	
**1,25(OH)_2_D_3_ (ng/mL)** ^**b**^	–	0.21	0.13	
**24,25(OH)_2_D_3_ (ng/mL)** ^**b**^	–	0.96	0.62	
**25(OH)D_3_/24,25(OH)_2_D_3_ ratio**	–	64.0	59.3	

Abbreviations: BSAP, bone specific alkaline phosphatase; eGFR, estimated glomerular filtration rate; FGF23, Fibroblast growthfactor 23; 25(OH)D, 25-hydroxyvitamin D; 1,25(OH)D₃, 25-hydroxyvitamin D; 24,25(OH)_2_D_3_, 24,25-dihydroxyvitamin D; PTH, parathyroid hormone; S-CTX, serum carboxy-terminal collagen crosslinks.

a Using immunochemiluminescent assay.

b Using mass spectrometry.

In case 1, additional vitamin D metabolite profiling was performed on plasma samples collected on September 2022 and May 2023. Vitamin D_3_, 25(OH)D_3_, 24,25(OH)_2_D_3_, and 1,25(OH)_2_D_3_ were measured by high-performance liquid chromatography–tandem mass spectrometry (HPLC–MS/MS). The 25(OH)D_3_/24,25(OH)_2_D_3_ ratio was calculated using concentrations expressed in the same units. Full details of the HPLC–MS/MS analytical method are provided in the Supplementary Materials.

### Bone Mineral Density (BMD)

BMD was assessed using DXA with a Hologic QDR-4500 densitometer (Hologic Inc.) at the LS (L1-L4, posterior–anterior projection), femoral sites, and the distal one-third radius. The coefficients of variation were 1.1% at the LS, 1.2% at the femoral neck and 1.4% at 1/3 distal radius. Vertebral fracture assessment (VFA) was carried out using lateral DXA scans of vertebrae spanning from T4 to L4.

### Genetic analysis

Genomic DNA was extracted from peripheral blood leukocytes according to standard procedures. The proband’s DNA was analyzed by next-generation sequencing (NGS) with a customized SureSelectXT (Agilent) panel targeting selected vitamin D-associated genes. Sequencing was performed on a MiSeq system (Illumina) using a paired-end protocol. Data were analyzed with the Variant Interpreter software (Illumina), and sequence alignment/review against the human reference genome were performed using Integrative Genomics Viewer. Direct Sanger sequencing (BigDye Terminator v3.1 Cycle Sequencing Kit, Life Technologies) was used to confirm variants identified by NGS and to test additional family members.

### Literature search

A literature search was performed in MEDLINE (via PubMed) and Scopus to identify relevant studies. Search terms included “CYP24A1 AND primary hyperparathyroidism” and “CYP24A1 AND hypercalcemia.” The last search was performed in December 2025. Reference lists were also screened to identify additional relevant reports. English-language full-text case reports and case series were included when they described patients with a *CYP24A1* variant and clinical, biochemical, imaging, surgical, and/or histological evidence of coexistent PHPT. Reports were included only when sufficient individual-level clinical and biochemical information was available. Reports of CYP24A1-related hypercalcemia without evidence of coexistent PHPT, and duplicate descriptions of the same patient were excluded. After duplicate removal, titles and abstracts were screened for relevance, followed by full-text assessment of potentially eligible reports. Data were extracted on age, sex, *CYP24A1* genotype and allelic status, serum calcium, phosphate, PTH and assay-specific reference ranges, renal manifestations, parathyroid findings, treatment, histology, and postoperative biochemical outcome. Seven publications describing 8 patients fulfilled the eligibility criteria and were included.

To explore whether previously proposed biochemical cut-offs could help to identify patients warranting further investigation for CYP24A1-related disease, serum calcium >9.6 mg/dL and a PTH index <0.315 were retrospectively applied to the present cases and to those included in the literature review.^5^ The PTH index was calculated as the measured PTH concentration divided by the assay-specific upper limit of normal.

## Case 1

In July 2018, a 26-yr-old man was referred to our outpatient clinic for evaluation of suspected PHPT incidentally identified during an occupational health assessment. Initial testing showed moderate hypercalcemia (12.3 mg/dL; reference range: 8.3-10.2) with an inappropriately normal PTH level (33.5 pg/mL; reference range: 15-65). His past medical history was remarkable for the presence of symptomatic recurrent bilateral nephrolithiasis beginning at 16 yr of age, while his father had experienced a single episode of renal colic. There was no family history of hypercalcemia, parathyroid disease or other endocrinopathy or neuroendocrine tumors. Physical examination was unremarkable.

Biochemical evaluation confirmed hypercalcemia with inappropriately normal PTH levels (Table 1). Serum creatinine was mildly elevated, with a reduced eGFR consistent with CKD stage G2. Serum 25(OH)D_3_ was within the normal range. Twenty-four hour urinary calcium excretion was elevated, and bone turnover markers were at the upper limit of the normal range (Table 1). Prolactin, growth hormone (GH), insulin-like growth factor I (IGF-I), thyroid-stimulating hormone (TSH), adrenocorticotropic hormone (ACTH), cortisol, insulin, gastrin, and glucose concentrations were within the normal range (data not shown). Renal ultrasound demonstrated bilateral nephrolithiasis with mild pelvicalyceal dilation. DXA and VFA showed normal BMD at LS, hip, and 1/3 distal radius and no vertebral fractures, respectively. Given the young age at diagnosis of PHPT, genetic testing using NGS panels of genes associated with familial PHPT (*MEN1*, *CASR*, *CDC73*, *CDKN1B*, *CDKN1A*, *CDKN2B*, *CDKN2C*, *GCM2,* and *AIP*) was performed in accordance with current guidelines^2^ and yielded negative results.

Neck ultrasound identified a 1.3-cm hypoechoic lesion posterior to the lower pole of the right thyroid lobe, suggestive of an enlarged right inferior parathyroid gland. Single-photon emission computed tomography (SPECT-CT) confirmed a concordant focus of uptake posterior to the right thyroid lobe. For the treatment of hypercalcemia, cinacalcet (30 mg twice daily) was initiated but discontinued after 2 wk due to gastrointestinal intolerance. In September 2018, he underwent right inferior PTx. Intraoperative PTH decreased from 88 to 27 pg/mL at 5 min indicating a successful PTx. Histopathological examination confirmed a chief cell parathyroid adenoma. On postoperative day 1, serum calcium remained elevated at 11.1 mg/dL. Although weekly serum calcium monitoring and a follow-up visit 1 mo after discharge were scheduled, the patient did not attend subsequent assessments. Because biochemical follow-up was unavailable during the subsequent 4 yr, it was not possible to determine whether hypercalcemia persisted after surgery or recurred following an undocumented period of normocalcemia.

He returned in September 2022 after incidental hypercalcemia detected on routine blood testing (10.8 mg/dL; reference range: 8.2-10.4) with PTH at the lower limit of normal (5.6 pg/mL; reference range: 4.6-58.1). Blood tests confirmed hypercalcemia (11.3 mg/dL), low PTH, elevated 25(OH)D_3,_ and 1,25(OH)_2_D_3_ (Table 1). Vitamin D metabolite profiling was also performed by HPLC–MS/MS and showed a 25(OH)D_3_ concentration of 61.6 ng/mL and a 24,25(OH)_2_D_3_ concentration of 0.96 ng/mL, corresponding to a 25(OH)D_3_/24,25(OH)_2_D_3_ ratio of 64.0 (Table 1).

No current or previous use of vitamin D supplements or vitamin D-containing preparations was documented. At dietary assessment, estimated daily calcium intake was approximately 360 mg. His renal function had declined (Table 1). He reported no recurrence of symptomatic nephrolithiasis. Renal ultrasound identified a 12-mm right renal calculus and multiple hyperechoic spots, suggestive of microlithiasis. DXA showed normal BMD. Bone turnover markers were within the reference range. Serum calcium screening in first-degree relatives (father, mother, and 2 sisters) was normal.

Given his young age, longstanding nephrolithiasis, persistent hypercalciuria, PTH-independent hypercalcemia with elevated vitamin D metabolite concentrations, and relatively high 25(OH)D_3_/24,25(OH)_2_D_3_ ratio, genetic testing of *CYP24A1* gene was performed. We identified a heterozygous pathogenic *CYP24A1* variant (c.1226T>C; p.Leu409Ser), also subsequently identified in his mother. This variant has been previously reported in the literature and represents the third most frequently reported variant.^4^

Following diagnosis, he was advised to limit sunlight exposure, maintain adequate hydration, and adopt a moderate dietary calcium intake. At last follow-up in May 2023, serum calcium remained mildly elevated but had decreased compared with September 2022, in parallel with declining 25(OH)D_3_ and 1,25(OH)_2_D_3_ levels and reduced 24-h urinary calcium excretion. Intact FGF23 was elevated (Table 1).

## Case 2

In January 2017, a 59-yr-old woman was referred following incidental biochemical findings suggestive of PHPT, with moderate/severe hypercalcemia (13.9 mg/dL; reference range: 8.2-10.6) and elevated PTH (119.5 pg/mL; reference range: 2.5-73). Her medical history included type 2 diabetes and hypertension, and she was receiving cholecalciferol (25 000 IU weekly). Family history was negative for hypercalcemia, parathyroid disease or other endocrinopathy, urolithiasis, or peptic ulcer disease in other family members.

At the time of our evaluation, physical examination was unremarkable.

Blood tests confirmed PTH-dependent hypercalcemia (Figure 1). The 24-h urinary calcium (224 mg/24 h), 25(OH)D_3_ concentrations (30.9 ng/mL), and bone turnover markers were within the normal range (Table 2). Abdominal CT scan did not reveal nephrolithiasis. BMD at LS, FN, TH, and 1/3 distal radius was normal, and VFA showed no vertebral fractures.

**Figure 1:**
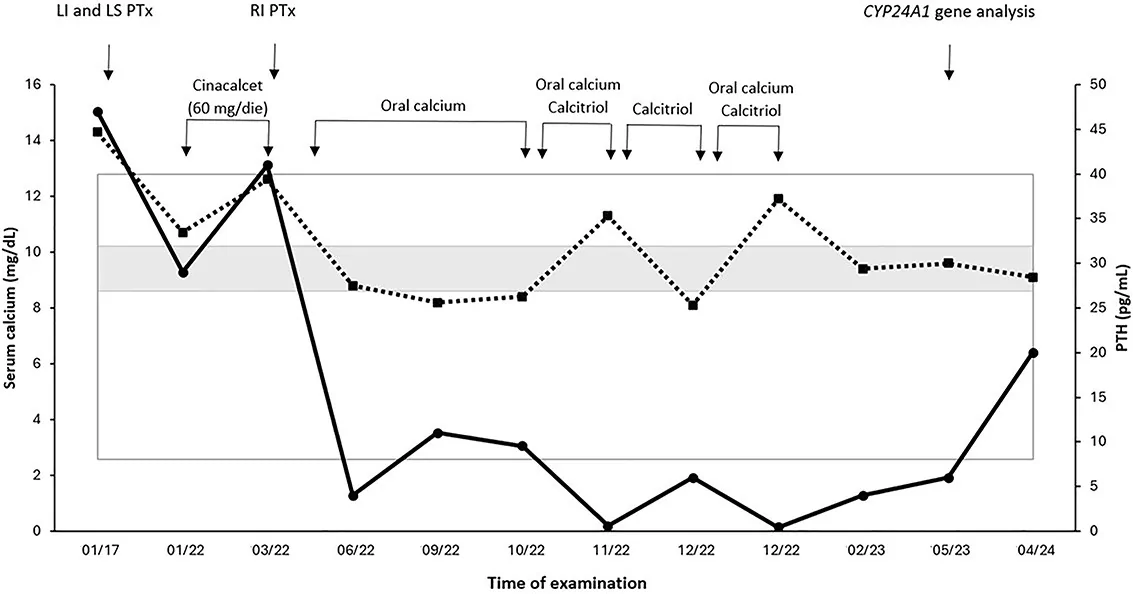
Evolution of serum calcium and PTH concentrations in case 2. The dotted line represents serum calcium and the solid line represents PTH. The light gray and white areas represent the normal ranges of serum calcium and PTH, respectively. The arrows indicate the time of surgery, medical treatment, and genetic testing. LI, left inferior; LS, left superior; PTx, parathyroidectomy; RI, right inferior.

**Table 2:** Biochemical parameters at baseline and after surgery of case 2.

Parameter	Pre first surgery (January 2017)	60 mo post first surgery (January 2022)	Pre second surgery (March 2022)	12 mo post second surgery (May 2023)	24 mo post second surgery (April 2024)	Reference range
**Ongoing daily therapy**	None	Cinacalcet 60 mg	Cinacalcet 60 mg	None	None	
**Serum calcium (mg/dL)**	14.3	10.7	12.6	9.6	9.1	8.6-10.2
**Ionized calcium (mmol/L)**	1.81	1.41	1.58	1.19	1.16	1.13-1.32
**PTH (pg/mL)**	47	29	41	6	20	8-40
**Phosphate (mg/dL)**	1.6	3.5	3.7	4.4	4.6	2.5-4.5
**Magnesium (mg/dL)**	1.6	1.0	1.6	2.1	1.9	1.7-2.5
**eGFR (mL/min/1.73 m** ^**2**^ **)**	75	45	37	40	36	
**Creatinine (mg/dL)**	0.86	1.33	1.54	1.45	1.56	0.7-1.2
**25(OH)D_3_ (ng/mL)**	30.9	30.1	39.4	33.4	20.6	20-100
**1,25(OH)**_**2**_ **D**_**3**_ **(pg/mL)**	–	–	–	21.2	34.1	20-79
**BSAP (mcg/L)**	19	17	18	11.3	19	2-28
**Osteocalcin (mcg/L)**	17.4	18.4	30.2	37.5	18.6	6.8-34
**S-CTX (mcg/L)**	0.360	0.125	0.125	0.186	0.193	0.034-1.037
**FGF23 (ng/L)**	–	–	–	303.8	225.8	23.2-95.4

Abbreviations: BSAP, bone specific alkaline phosphatase; eGFR, estimated glomerular filtration rate; 25(OH)D_3_, 25-hydroxyvitamin D; S-CTX, serum carboxy-terminal collagen crosslinks.

Neck ultrasound showed a 1.8-cm hypoechoic lesion consistent with an enlarged left superior parathyroid gland, together with a large multinodular goiter. Single-photon emission CT revealed focal early tracer uptake at the superior pole of the left thyroid lobe. In February 2017, she underwent combined PTx and total thyroidectomy. At surgery, both the left superior and left inferior parathyroid glands appeared enlarged and were removed. Intraoperative PTH decreased from 175 to 115 pg/mL at 5 min (34% reduction). Histopathological examination showed a chief-cell parathyroid adenoma in the left superior gland and a lipoadenoma in the left inferior gland. At discharge, serum calcium was 10.6 mg/dL. Outpatient follow-up was scheduled, but the patient preferred to continue further investigations with the referring endocrinologist closer to home.

In June 2021, she presented to the emergency department with prolonged nausea, vomiting, and severe hypercalcemia (19.8 mg/dL) with elevated PTH levels (226 pg/mL). Management included hemodialysis and intravenous zoledronic acid. Renal ultrasound revealed bilateral nephrolithiasis, while dual-phase ^99^mTc-sestamibi planar scintigraphy was negative. At discharge, calcium was 10.2 mg/dL and PTH 84.8 pg/mL. Cinacalcet 30 mg/d was started.

In November 2021, the patient was again referred to the emergency department given recurrent nausea, vomiting, persistent hypercalcemia, and high PTH levels (calcium 13.8 mg/dL; PTH 124 pg/mL). Intravenous zoledronic acid was administered, resulting in normalization of serum calcium within 2 wk; cinacalcet was then increased to 60 mg/d.

In January 2022, the patient returned to our outpatient clinic. Serum calcium was elevated despite cinacalcet therapy (10.7 mg/dL on 60 mg/d) with inappropriately normal PTH concentrations (Figure 1). After 2 mo, in March 2022, serum calcium further increased (12.6 mg/dL) while urinary calcium, bone turnover markers (Table 2), and BMD at the 3 sites remained within normal ranges. Neck ultrasound revealed a hypoechoic lesion in the right lower region, whereas SPECT/CT was negative. Genetic testing performed to investigate multiglandular PHPT was negative. Cinacalcet was increased to 120 mg/d, and in May 2022 she underwent right inferior PTx with cervical exploration. Intraoperative PTH decreased from 169 to 60 pg/mL at 5 min. Histology showed a chief-cell parathyroid adenoma. Postoperatively, serum calcium normalized to 9.7 mg/dL, and calcium carbonate (1000 mg/d) was initiated. At 1 mo (June 2022), serum calcium was 8.8 mg/dL with undetectable PTH while receiving calcium carbonate 1000 mg/d (Figure 1). In view of the low serum calcium and low PTH levels detected during follow-up, calcitriol 0.25 μg/d was added to ongoing calcium carbonate supplementation. However, during subsequent biochemical follow-up, serum calcium again increased, ranging from 10.2 to 11.9 mg/dL while the patient was receiving both calcium carbonate and calcitriol; both drugs were therefore discontinued (Figure 1).

In May 2023, serum calcium was 9.6 mg/dL, and PTH was lower than the limit of normal (Figure 1). Abdominal ultrasound showed bilateral nephrocalcinosis. Renal function was reduced (eGFR 40 mL/min/1.73 m^2^), and FGF23 was elevated (303.8 ng/L), while 25(OH)D_3_ and 1,25(OH)_2_D_3_ were within reference ranges (33.4 and 21.2 pg/mL, respectively) (Table 2).

Given the marked variability in calcium balance, particularly in relation to calcium and calcitriol exposure, genetic testing using a stored blood sample was performed and compound heterozygous *CYP24A1* variants were identified: c.428_430del (p.Glu143del) and c.245A>G (p.Gln82Arg). Screening for hypercalcemia in first-degree relatives was not performed because the patient’s parents, and brother were deceased, and her 2 sons declined testing. Following diagnosis, she was advised to limit sun exposure, maintain adequate hydration, and follow a moderate calcium diet. At the most recent evaluation (April 2024), serum calcium and PTH were normal (Figure 1), whereas phosphate was at the upper limit of normal (4.6 mg/dL). Abdomen ultrasound confirmed persistent bilateral nephrocalcinosis with loss of corticomedullary differentiation. Renal function had further declined (eGFR 36 mL/min/1.73 m^2^), and FGF23 remained elevated (225.8 ng/L), with normal vitamin D metabolites [25(OH)D_3_, 20.6 ng/mL; 1,25(OH)_2_D_3_, 34.1 pg/mL]. Unfortunately, it was not possible to perform vitamin D metabolite profiling by HPLC–MS/MS, as the patient was unable to return to our hospital to provide an additional blood sample.

## Discussion

Hypercalcemia poses a diagnostic challenge because biochemical patterns may overlap across etiologies. A pragmatic first step is to classify it as PTH-dependent or PTH-independent based on circulating PTH levels.^2^ In PHPT, hypercalcemia is typically associated with non-suppressed PTH; however, this framework can be confounded when *CYP24A1* variants coexist. PHPT is often detected incidentally, although nephrolithiasis, nephrocalcinosis, and fractures may occur.^8^ In both of our patients, hypercalcemia was incidentally detected, but calcium levels were higher than those usually reported in unselected PHPT cohorts.^9^ Clinical suspicion of CYP24A1-related disease should be heightened in patients presenting at a young age, particularly in the presence of a family history of hypercalcemia or nephrolithiasis, recurrent nephrolithiasis or nephrocalcinosis, relatively high 25(OH)D_3_ concentrations in the setting of hypercalcemia, or an increased 25(OH)D_3_/24,25(OH)_2_D_3_ ratio.^3^^,^^4^^,^^10^ A marked worsening of hypercalcemia during pregnancy or lactation has also been described and may represent an additional diagnostic clue.^10^

In case 1, the initial presentation was consistent with PHPT as supported by moderate hypercalcemia, inappropriately normal PTH levels, concordant imaging, and adenoma histology. However, he later developed hypercalcemia with suppressed PTH levels. Elevated 25(OH)D_3_ and 1,25(OH)_2_D_3_ in the absence of supplementation prompted further work-up. Although the highest 25(OH)D_3_ concentrations were measured in summer or early autumn, habitual sunlight exposure was not formally quantified; therefore, a contribution of seasonal ultraviolet exposure cannot be excluded. However, the relatively high 25(OH)D_3_ concentrations, particularly in the presence of concomitant hypercalcemia and suppressed PTH levels, contributed to the suspicion of impaired vitamin D catabolism which was confirmed by HPLC–MS/MS vitamin D profiling. Genetic testing revealed a heterozygous *CYP24A1* pathogenic variant (p.Leu409Ser), consistent with partial enzyme deficiency. This finding raises the possibility that impaired CYP24A1-mediated vitamin D catabolism contributed to the postoperative biochemical phenotype. However, given the monoallelic genotype, the magnitude of this contribution cannot be precisely established. This case suggests that CYP24A1 defects may be suspected in patients with suppressed PTH despite apparently successful parathyroid surgery.

Only one previous case of PHPT in a heterozygous *CYP24A1* carrier has been reported.^11^ In that patient, hypercalcemia persisted despite hydration, bisphosphonates, and high-dose cinacalcet, and normalized only after PTx, with subsequent PTH suppression.^11^ Compared with that case, our heterozygous carrier also had nephrolithiasis and reduced renal function, features less common in heterozygous carriers, who usually show a milder phenotype with lower rates of nephrocalcinosis and clinically significant nephrolithiasis.^3^^,^^7^ Additional precipitating factors, including vitamin D exposure or PHPT itself, may unmask or amplify the defect, producing a more overt biochemical and clinical phenotype.^7^^,^^12^^,^^13^

Case 2 had a more complicated clinical course, with multiglandular PHPT requiring 2 parathyroid surgeries. Calcium fluctuations, particularly after exposure to calcitriol, together with nephrocalcinosis and progressive renal impairment, suggested PTH-independent hypercalcemia. The p.Glu143del variant found in our patient has previously been reported in a woman with incidentally detected hypercalcemia and CKD stage 3A. In that case calcium normalized after PTx, but hypercalcemia recurred with suppressed PTH after cholecalciferol administration, prompting genetic testing and confirmation of a pathogenic *CYP24A1* variant.^14^ Of note, 1,25(OH)_2_D_3_ was in the low-normal range (21.2 pg/mL) in May 2023 despite the biallelic *CYP24A1* genotype. This finding may reflect reduced calcitriol production related to impaired renal function, markedly elevated FGF23 concentrations, and low PTH levels following parathyroid surgery, all of which may counterbalance the reduced vitamin D catabolism associated with CYP24A1 deficiency.

Measurement of 24,25(OH)_2_D_3_ together with 25(OH)D_3_, allowing calculation of the 25(OH)D_3_/24,25(OH)_2_D_3_ ratio, represents the most informative biochemical approach for identifying impaired CYP24A1-mediated vitamin D catabolism and selecting patients for molecular testing. In Case 1, the 25(OH)D_3_/24,25(OH)_2_D_3_ ratio was increased at 2 postoperative assessments (64.0 and 59.3), providing biochemical support for impaired CYP24A1-mediated vitamin D catabolism. Although below the >80 threshold commonly associated with biallelic deficiency, ratios between 25 and 80 may occur in *CYP24A1* variant carriers, particularly in monoallelic disease.^5^^,^^15^ Thus, together with the heterozygous genotype, these findings support partial impairment of vitamin D catabolism, while the extent of its contribution to the postoperative phenotype remains uncertain. Although metabolite profiling may represent a clinically useful step before genetic investigation, measurement of 24,25(OH)_2_D_3_ requires validated HPLC–MS/MS and remains available mainly in specialized laboratories. Its accessibility relative to molecular testing may therefore vary across healthcare settings.

The literature review identified eight cases of CYP24A1-related disease and PHPT. Seven patients carried biallelic *CYP24A1* variants (3 homozygous and 4 compound heterozygous), while one harbored a heterozygous variant (Table 3).^11^^,^^14^^,^^16–20^ Although biallelic variants were originally associated with a severe childhood-onset phenotype, adult presentations are increasingly recognized, and the cases identified in our review further support substantial phenotypic variability.^4^^,^^21^

**Table 3:** Summary of clinical cases with primary hyperparathyroidism associated with *CYP24A1* variant reported in the literature.

Reference	*CYP24A1* variant	Genotype	Renal involvement	Serum calcium (mg/dL)	Serum phosphate (mg/dL)	PTH/ULN	Management		Outcome
							Medical therapy of hypercalcemia	Type of Surgery	
**Helmuth et al.** ^16^	p.Arg396Trp	Homo	Nephrolithiasis Nephrocalcinosis CKD stage 2	14.0 (8.4-10.2)	3.0 (2.3-4.8)	0.68	Bisphosphonates Cinacalcet	First surgery: sPTx Second surgery: TPTx	Normocalcemia PTH/ULN 0.22
**Loyer et al.** ^17^ **Case 1**	p.Cys380Arg p.Leu409Ser	Comp Het	Nephrolithiasis Nephrocalcinosis CKD stage 3a	13.9 (8.8-10.2)	2.8 (2.5-4.5)	1.42	Cinacalcet	sPTx	Moderate hypercalcemia PTH/ULN 0.95
**Loyer et al**.^17^ **Case 2**	p.Leu335Profs^*^11 p.Arg396Gln	Comp Het	Nephrolithiasis Nephrocalcinosis CKD stage 3a	11.5 (8.6-10.2)	2.5 (2.5-4.5)	0.42	None	sPTx	Mild hypercalcemia PTH/ULN 0.52
**David et al.** ^19^	p.Arg396Trp	Homo	Nephrolithiasis Nephrocalcinosis CKD stage 3b	12.2 (8.6-10.2)	2.3 (2.6-4.7)	0.83	Bisphosphonates Cinacalcet	TPTx	Normocalcemia PTH/ULN 0.04
**Collins et al.** ^18^	p.Arg396Trp	Homo	CKD stage 2	12.3 (8.6-10.6)	3.1 (2.5-4.3)	0.33	None	First surgery: sPTx Second surgery: sPTx	Mild hypercalcemia PTH/ULN 0.04
**Liu et al.** ^11^	p.Pro392Argfs^*^9	Het	None	13.8 (8.4-10.2)	2.1 (2.8-4.5)	0.70	Bisphosphonates Cinacalcet	sPTx	Normocalcemia PTH/ULN 0.16
**Leszczynska et al.** ^14^	p.Arg396Trp p.Glu143del	Comp Het	CKD stage 3a	12.0 (8.8-10.2)	0.8 (2.5-4.5)	0.42	None	sPTx	Normocalcemia PTH/ULN 0.23
**Zhang et al.** ^20^	p.Arg396Trp p.Trp210Arg	Comp Het	Nephrolithiasis	12.9 (8.6-10.2)	–	0.88	None	sPTx	Mild hypercalcemia PTH/ULN 0.42
**Present study** **Case 1**	p.Leu409Ser	Het	Nephrolithiasis CKD stage 2	12.5 (8.6-10.2)	2.1 (2.5-4.5)	0.60	Cinacalcet	sPTx	Mild hypercalcemia PTH/ULN 0.23
**Present study** **Case 2**	p.Glu143del p.Gln82Arg	Comp Het	Nephrolithiasis CKD stage 2	14.3 (8.6-10.2)	1.6 (2.5-4.5)	1.18	Bisphosphonates Cinacalcet	First surgery: sPTx Second surgery: sPTx	Normocalcemia PTH/ULN 0.50

Abbreviations: Comp Het, compound heterozygous; Het, heterozygous; Homo, homozygous; PTH/ULN, ratio of measured PTH to the assay-specific upper limit of normal (ULN); sPTx, selective parathyroidectomy; TPTx, total parathyroidectomy.

Mild and moderate hypercalcemia were defined according to the authors’ reports or postoperative serum calcium values when available. Normal ranges for serum calcium and phosphate are shown in brackets.

Biochemically, the mean PTH-to-upper-limit-of-normal across cases, including our patients, was 0.75, suggesting that PHPT was usually suspected because of inappropriately normal rather than overtly elevated PTH levels. A key feature was the discordance between calcium and PTH, with a median serum calcium of 12.7 mg/dL (IQR 12.3-13.9) and median phosphate 2.3 mg/dL (IQR 2.1-2.8). In classical PHPT, calcium and PTH typically rise in parallel, reflecting Parfitt’s “rightward shift” of the calcium–PTH set-point.^22^ In CYP24A1-related hypercalcemia, PTH elevations are often blunted/normal despite moderate-severe hypercalcemia, likely due to calcitriol accumulation and the modulation by FGF23 activity. Exploratory application of the previously proposed biochemical thresholds by our group showed that, before PTx, both patients described herein and all patients reported in the literature^11^^,^^14^^,^^16–20^ fulfilled the serum calcium criterion (>9.6 mg/dL), whereas none had a PTH index <0.315, likely because active PHPT prevented appropriate PTH suppression. After PTx, serum calcium was >9.6 mg/dL in 7 of 10 patients (70%), the PTH index was <0.315 in 6 of 10 (60%), and both thresholds were fulfilled in 4 of 10 (40%). Notably, Case 1 fulfilled both criteria, whereas Case 2 fulfilled the PTH-index criterion but not the calcium criterion, as serum calcium was exactly 9.6 mg/dL.

For exploratory comparison, the same PTH threshold was examined in a series of unselected consecutive 21 patients referring to our outpatient’s clinic with biochemically persistent (*n* = 14) or recurrent (*n* = 7) PHPT after PTx. Median serum calcium was 10.7 mg/dL (IQR 10.6-10.8) and median PTH index was 0.98 (IQR 0.88-1.17); none of these patients had a PTH index <0.315. These findings are consistent with the diagnosis of PHPT and suggest that a low PTH index may be a useful marker for raising suspicion of CYP24A1-related disease when postoperative hypercalcemia is no longer PTH-dependent.

Thus, in summary, taking these exploratory comparisons together, we found that these cut-offs would have identified only a subset of patients with coexistent PHPT and *CYP24A1* variants. Moreover, they may be particularly useful in patients with postoperative hypercalcemia or high-normal calcium concentrations accompanied by disproportionately low PTH levels, in whom they could prompt vitamin D metabolite profiling and, when supported by the biochemical findings, molecular testing of *CYP24A1*. However, failure to meet both thresholds does not exclude CYP24A1-related disease. Because the cut-offs were derived from patients with biallelic *CYP24A1* variants without coexistent PHPT and have not been validated after PTx, they should therefore be regarded as exploratory screening aids rather than diagnostic criteria.

Renal involvement is central in both PHPT and CYP24A1-related disease. Persistent elevation of 1,25(OH)_2_D_3_ in CYP24A1 deficiency increases intestinal calcium absorption and causes chronic hypercalciuria,^23^^,^^24^ leading to progressive renal impairment. In reported cases of PHPT associated with *CYP24A1* variants, 90% had at least one renal complication (*n* = 9): impaired renal function in 80% (*n* = 8), nephrolithiasis in 70% (*n* = 7), and nephrocalcinosis in 40% (*n* = 4) (Table 3).^3^^,^^4^^,^^7^ However, these findings are derived from a very small number of published case reports and are likely influenced by preferential reporting of more severe or clinically unusual phenotypes. Therefore, they should not be interpreted as estimates of the prevalence or risk of renal involvement in patients with coexistent PHPT and *CYP24A1* variants. Although the 2 conditions may plausibly contribute to renal morbidity through partly overlapping mechanisms, the available data do not establish a synergistic effect.

Medical management is challenging. Cinacalcet may lower serum calcium in PHPT through activation of the calcium-sensing receptor (CaSR) on parathyroid cells and suppression of PTH secretion. In patients with coexistent PHPT and CYP24A1-related disease, however, its efficacy may vary because hypercalcemia may also be sustained by impaired vitamin D catabolism and increased 1,25(OH)_2_D_3_-mediated intestinal calcium absorption (Table 3).^11^^,^^16^^,^^17^^,^^19^ Notably, Mohan et al. reported normalization of serum calcium with cinacalcet in a patient with biallelic *CYP24A1* variants, low PTH, and persistently inappropriate 1,25(OH)_2_D_3_ concentrations. A possible PTH-independent mechanism involving activation of intestinal CaSR and attenuation of 1,25(OH)_2_D_3_-mediated calcium absorption has been proposed, although this remains speculative.^25^

Bisphosphonates can provide temporary calcium reduction by inhibiting bone resorption but do not correct sustained intestinal calcium influx.^11^^,^^16^^,^^19^ Azoles or rifampin have been proposed to reduce 1,25(OH)_2_D_3_ synthesis or enhance catabolism. Ketoconazole and fluconazole may reduce 1,25(OH)_2_D_3_ production by inhibiting cytochrome P450 enzymes involved in 1α-hydroxylase activity, whereas rifampin induces CYP3A4, providing an alternative pathway for the inactivation of 25(OH)D_3_ and 1,25(OH)_2_D_3_. However, evidence for these treatments is limited to individual cases or small series, particularly in patients with coexistent PHPT.^13^^,^^26^^,^^27^

PTx remains the definitive treatment for the PHPT component, but hypercalcemia may persist when CYP24A1 deficiency coexists. Long-term care should include avoidance of vitamin D supplements, moderation of dietary calcium, limited sun exposure, and high fluid intake.^7^^,^^12^^,^^13^ However, it should be emphasized that an excessive calcium restriction (<400 mg daily) should be avoided, as low calcium intake may increase intestinal oxalate absorption and urinary oxalate excretion, potentially offsetting the expected benefit on hypercalciuria.^28^ Across the 10 cases, all patients underwent PTx, for a total of 13 procedures: selective PTx in 11 and total PTx in 2. Outcomes were favorable overall with sustained normocalcemia in 5, residual hypercalcemia in 5 (mild in 4, moderate in 1) (Table 3).

The coexistence of PHPT and *CYP24A1* variants raises pathophysiological questions. Although the association may be coincidental, given the prevalence of PHPT,^9^ potential biological links between altered vitamin D metabolism and parathyroid physiology can be considered as hypothesis-generating mechanisms: (1) phosphate may influence parathyroid cell biology. Experimental studies, predominantly conducted in CKD models, suggest that increased phosphate levels may promote parathyroid cell proliferation and downregulate CaSR and Vitamin D receptor expression, thereby attenuating the inhibitory control of PTH secretion.^29^^,^^30^ If CYP24A1-related disease increases phosphate absorption, it might contribute to this pathway. However, in our patient serum phosphate levels were within the normal range, possibly due to the opposite effects of the 2 conditions: PHPT promotes renal phosphate wasting, whereas CYP24A1 deficiency increases intestinal phosphate absorption; (2) recurrence of specific *CYP24A1* variants among the reported cases is noteworthy, including p.Arg396Trp in 5 patients and p.Leu409Ser and p.Glu143del in 2 patients each. This distribution may simply reflect relatively frequent pathogenic alleles, population effects, or reporting bias, and the small number of cases does not support an association between individual variants and PHPT susceptibility or severity^4^; (3) elevated FGF23 suggests broader mineral dysregulation. Physiologically, FGF23 limits 1,25(OH)_2_D_3_ accumulation by suppressing CYP27B1 and stimulating CYP24A1 expression.^31^ In patients with *CYP24A1* loss-of-function variants, increased FGF23 levels may therefore represent an adaptive response to chronically elevated 1,25(OH)₂D_3_ concentrations. However, in the setting of CKD, the regulatory effects of FGF23 on PTH may be attenuated because of parathyroid resistance related to reduced FGFR1/Klotho signaling.^32^^,^^33^ Thus, the available data do not allow us to determine whether FGF23 contributed to the development or severity of PHPT.

This report is limited by its retrospective description of only 2 patients. Comprehensive vitamin D metabolite profiling was available only for Case 1. In Case 2, 24,25(OH) _2_D_3_ measurement and calculation of the 25(OH)D_3_/24,25(OH)_2_D_3_ ratio could not be performed, and postoperative follow-up was incomplete in Case 1.

In conclusion, these cases illustrate that *CYP24A1* variants may coexist with PHPT and complicate the interpretation of persistent or recurrent hypercalcemia after PTx. When postoperative hypercalcemia is accompanied by suppressed PTH, hypercalciuria, nephrolithiasis or nephrocalcinosis, and relatively high vitamin D metabolite concentrations, investigation of impaired vitamin D catabolism should be considered. Whenever available, measurement of 24,25(OH)_2_D_3_ and calculation of the 25(OH)D_3_/24,25(OH)_2_D_3_ ratio may provide important biochemical support before or alongside molecular testing. Nevertheless, the phenotypic contribution of an identified *CYP24A1* variant, particularly a monoallelic variant, should be interpreted cautiously. Whether impaired CYP24A1 activity modifies the clinical expression of PHPT remains uncertain and requires further investigation.

## Supplementary Material

Supplementary_material_ziag164

## Data Availability

The data cannot be shared publicly due to privacy issues.
