# Supplementary material for: Co-occurrence of pathogenic CYP24A1 gene variants and primary hyperparathyroidism: a diagnostic challenge
Source: JBMR Plus. 2026 Oct 9;10(11):ziag164. doi: 10.1093/jbmrpl/ziag164 (PMC13695950; doi:10.1093/jbmrpl/ziag164)
Supplement: Supplementary file 1 [file supplementary_material_ziag164.docx]

**High-Performance Liquid Chromatography-Tandem Mass Spectrometry (HPLC-MS/MS) method for Vitamin D metabolites simultaneous measurement**

The HPLC-MS/MS instrumentation consisted of a UHPLC system (Agilent 1290 Infinity), coupled to a hybrid triple quadrupole/linear ion trap mass spectrometer Sciex QTrap 6500+, equipped with an electrospray source (ESI). The UHPLC device made use of a Waters BEH Phenyl column (Waters, Milford, MA, USA), supplied with water and methanol, both containing 0.5 mM methylamine. The detection method for 25(OH)D_3_, 24,25(OH)_2_D_3_, vitamin D_3_ and their respective internal standards was based on multiple reaction monitoring (MRM), while 1,25(OH)_2_D_3_ and its respective IS were acquired in MS^3^ (MS/MS/MS) with zero energy for the second fragmentation step. A validation protocol was defined using the EMA guidelines (European Medicines Agency, Guideline Bioanalytical method validation)^1^. After collection, plasma samples were stored at -20°C and, before analysis, were thawed at room temperature under gentle shaking. In microcentrifuge tubes, an aliquot of plasma samples or calibrators was mixed with acetonitrile containing 0,1% formic acid, and a proper amount of internal standards. The obtained mixture was vortexed, centrifuged, and the supernatant was evaporated under a gentle stream of nitrogen. Then, water was added to the dried samples and liquid-liquid extraction was performed by adding a hexane/dichloromethane (1:1, v/v). The upper organic phase was dried under nitrogen, and the dry residue was reconstituted and derivatized with PTAD (4-phenyl-1,2,4-triazoline 3,5-dione) and then incubated at room temperature in the dark. The reaction was stopped by adding a quench solution (water + 0.1% formic acid) and vortexing, and then each sample was evaporated under a gentle stream of N_2_. The dry residues were reconstituted with 50:50 (v/v) water/methanol solution containing 0.5 mM methylamine.

**Reference**

1. International Council for Harmonisation of Technical Requirements for Pharmaceuticals for Human Use (ICH). ICH guideline M10 on bioanalytical method validation – Step 5. EMA/CHMP/ICH/172948/2019. Amsterdam: European Medicines Agency; 2022
